# Cell Suspension of the Tree Fern *Cyathea smithii* (J.D. Hooker) and Its Metabolic Potential During Cell Growth: Preliminary Studies

**DOI:** 10.3390/ijms262311683

**Published:** 2025-12-02

**Authors:** Jan J. Rybczyński, Łukasz Marczak, Katarzyna Skórkowska-Telichowska, Maciej Stobiecki, Jan Szopa, Anna Mikuła

**Affiliations:** 1 Polish Academy of Sciences Botanical Garden—Center for Biological Diversity Conservation in Powsin, 2 Prawdziwka Str., 02-973 Warsaw, Poland; 2 Institute of Bioorganic Chemistry, Polish Academy of Sciences, 12/14 Noskowskiego Str., 61-704 Poznań, Poland; lukasmar@ibch.poznan.pl (Ł.M.); ibch@ibch.poznan.pl (M.S.); 3 Medical Faculty, Wrocław University of Science and Technology, pl. Grunwaldzki 11, 51-377 Wrocław, Poland; katarzyna.skorkowska-telichowska@pwr.edu.pl; 4 Department of Endocrinology, Jerzy Gromkowski Regional Specialist Hospital, 5 Koszarowa Str., 51-149 Wrocław, Poland; 5 Independent Researcher, 51-148 Wrocław, Poland; szopa@ibmb.uni.wroc.pl

**Keywords:** chemical analysis, cell aggregates, flavonoids, gas chromatography, liquid chromatography, mass spectrometry, primary and secondary metabolites

## Abstract

The purpose of this study was to present a chemical analysis of the metabolome of cell aggregates of the tree fern *Cyathea smithii* (J.D. Hooker) cell suspension culture. The LC/MS and GC/MS techniques were used for identification of metabolites. The kinetics of fresh weight, dry weight, and ash content showed 3.5-fold increases during 15-day-long culture. The analysis demonstrated high metabolic activity of cultured cells. In total, 160 metabolites from primary and secondary metabolism and almost 2000 compounds of unknown identity were identified. Three flavonoids—the chalcone isookanin [(2S)-2-(3,4-dihydroxyphenyl)-7,8-dihydroxy-2,3-dihydrochromen-4-one], a methoxy derivative of the flavone gardenin B (5-Hydroxy-2-(4-methoxyphenyl)-6,7,8-trimethoxy-4H-1-benzopyran-4-one), and the isoflavone tectoridin (4′,5-Dihydro-6-methoxy-7-(O-glucoside)isoflavone)—had not been previously detected in the cell culture of *C. smithii*. Principal component analysis revealed five distinct groups of samples; groups 4 and 5 showed the greatest similarity and corresponded to cultures on days 12 and 15, respectively. The number of differentiating compounds was 75, indicated by a heatmap showing positive and negative correlations between the days of culture. The studies described in this paper are crucial for further identification of metabolites and establishing the relationship between the metabolic composition of tree fern cells in culture and their biological activity, assessed by physiological parameters. By determining the relationship between the chemical composition of cells and their growth from culture initiation to senescence, we will provide a more complete picture of the potential for environmental factors to regulate this relationship. Based on previous studies, environmental stimuli such as electromagnetic fields or light of different wavelengths can result in altered growth physiology and cell mass, as well as metabolite diversification and accumulation. The research results presented in this paper provide a foundation for further studies aimed at predicting and regulating the productivity of *C. smithii* cells in suspension culture and elucidating the significance of tree fern-derived metabolic products in human cell biology, particularly in thyroid cells.

## 1. Introduction

Ferns are one group of plants whose metabolic products are of interest to humans as sources of food and medicine [1,2]. They are renowned as traditional medicinal plants [2], with most attention focused on their radical-scavenging, antioxidant, and anticancer activities [3,4]. Only a few tree fern species have been investigated for this purpose to date. In the genus *Cyathea*, which is the largest among tree ferns (about 470 species), only about 20 species have been studied [5]. Pharmacological value has only been described for species traditionally used by indigenous peoples in the western and southeastern hemispheres. The analyzed plant material usually originates from young leaves [6,7,8], the caudex, leaves, fronds [9,10], and whole plants [11]. Tissue cultured in liquid medium under in vitro conditions has not been examined in this context, apart from our earlier studies on *Cyathea smithii* [12] and *C. delgadii* [13]. The first investigation of secondary metabolite constituents in the genus *Cyathea* was published in 1955 [14]. The researchers examined leaves of *Cyathea boninshimensis* to provide a comprehensive survey of the distribution of flavones, flavanols, and flavonones. The most recent review of the literature examining the potential of this genus as a source of bioactive molecules was conducted in 2022 [5]. Based on these findings, phenolic compounds were identified as the dominant bioactive components. Flavonoids are secondary metabolites found in all parts of living plants [15,16]. Under in vitro conditions, they are freely released from actively dividing cells into the liquid medium of the cell suspension in response to stress [17]. The intensive yellow coloration of the culture medium of the *Cyathea delgadii* cell suspension indicated active secondary metabolism and intensive flavonoid production [13] similar to that observed in the climbing perennial fern *Lygodium flexuosum* and the scrambling fern *Ampelopteris prolifera* [18].

*Cyathea smithii* is a medium-sized tree fern that can produce a slender trunk up to 8 m tall, although it is usually considerably shorter [19]. It has a spreading crown of flat, tripinnate fronds on profusely scaly stalks. The stalks persist on the trunk and can form a skirt below the crown. *Cyathea smithii* is native to cool mountain and lowland forests on the North and South Islands of New Zealand as well as Stewart Island, the Chatham Islands, and the subantarctic Auckland Islands far to the south, making it the world’s southernmost-occurring tree fern. Well-adapted to the cool and humid environment of its habitat, it grows poorly in hot climates and is less tolerant of higher temperatures than other tree ferns from comparable habitats, such as *Dicksonia antarctica*. *Cyathea smithii* tolerates moderate freezes and is one of the hardiest tree ferns [19,20]. In vitro culture is the only known method for establishing a plant multiplication system for this species [21]. In the presence of growth hormones, an apical dome appeared to be the source of meristematically active cells capable of proliferating and regenerating plants [22]. The highest number of regenerants was obtained on an agar medium supplemented with 5.0 µM TDZ and 0.25 µM NAA. The nuclear DNA content was assessed and ranged from 4.74 to 4.78 pg DNA in gametophytes and from 8.45 to 9.13 pg in sporophytes [22]. A cell suspension derived from callus tissue of root explants was cultured in ½ MS medium supplemented with 9.05 µM 2,4-D + 0.88 µM BAP [12]. Microscopic analysis of the cells helped elucidate the changes occurring during the 2-week subculture period. The suspension showed high physiological activity, as indicated by growth kinetics and the intensive production of ethylene and quercetin. Additionally, the post-culture liquid medium exhibited bactericidal activity against microaerophilic Gram-positive bacteria.

The advantage of a suspension culture system is that it provides an unlimited supply of plant material over long periods and thus preserves the plant gene pool under stable environmental conditions. Regular and precise subculturing of the cell suspension ensures the homogeneity of the experimental material. This paper presents the results of the continuation of earlier studies on *C. smithii* cells, which initially focused only on the structure and physiology of cell aggregates [12]. In contrast, the present study evaluates the ability of these aggregates to produce metabolites, with particular attention paid to secondary metabolites, especially flavonoids with diverse phenolic structures.

## 2. Results

### 2.1. Kinetics of Established Cell Suspension

**Figure 1:**
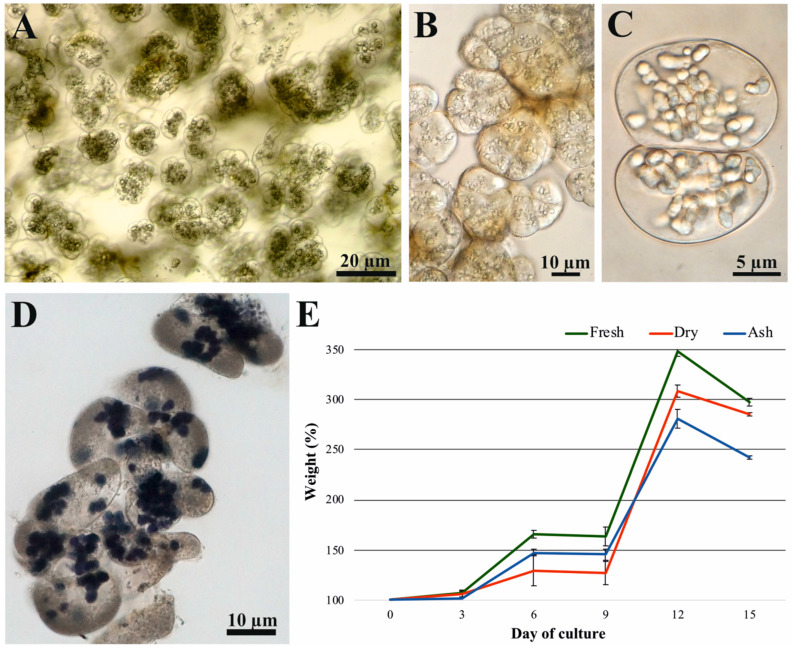
Cellular structure and the kinetics of fresh weight, dry weight, and ash content (%) of the *Cyathea smithii* cell suspension cultured in the presence of ½ MS medium supplemented with 9.05 µM 2,4-D + 0.88 µM BAP + 2.0% sucrose. (**A**) General view of cell aggregates (live specimen). (**B**) Regular cell divisions initiating the formation of cell aggregates (live specimen). (**C**) Twin cells observed in Nomarsky contrast showing very rich cytoplasm and thin cell walls (live specimen). (**D**) Squashed specimen (fixed tissue) indicating intensive starch grain formation stained by IKI and richness of the cytoplasm. (**E**) The content of fresh weight, dry weight, and ash (%) during 15-day-long culture (on day 0, 3rd, 6th, 9th, 12th, and 15th of culture).

The suspension culture, maintained in 250 mL conical flasks, comprised mostly cell aggregates (Figure 1A) and, to a lesser extent, individual cells. Figure 1B,C confirms the dividing and meristematic character of the cells with abundant cytoplasm, thin cell walls, and small vacuoles. The presence of dividing cells indicates dynamic growth of the suspension (Figure 1C). Most cells accumulated starch, visible as I-KI-stained starch grains (Figure 1D). The presence of BAP and 2,4-D stimulated cell proliferation in the suspension. The cell suspension exhibited growth characteristics typical for this culture type when initiated with 3 g of tissue in 80 mL of medium. Figure 1E shows fresh, dry, and ash weight growth curves of the established suspension culture. The three indicators followed similar sigmoidal growth patterns. The suspension reached its maximum growth on day 12 of culture.

### 2.2. Biochemical Analysis of Cell Aggregates

The biochemical analysis of the cell aggregates of the 15-day culture revealed 9 groups of organic compounds, each containing a different number of metabolites: acids—42, polyhydric alcohols—14, amino acids—11, amines—11, nucleosides—2, phosphates—12, saccharides—32, flavonoids—18, and others—14. Additionally, nearly 2000 compounds of unknown identity were detected.

*Organic acids*

Acids constituted the largest group (Table S1). However, they were very diverse and represented different metabolic pathways. The list of acids includes sugar acids, fatty acids, mono-, di-, and tricarboxylic acids, unsaturated acids, polyhydroxy acids and hydroxy acids, phenolic acids, keto acids, and vitamins. Some of these acids are intermediates of the tricarboxylic acid (TCA) cycle.

*Saccharides*

The second largest group consisted of saccharides (32 compounds, Table S2). The rich sugar metabolism of the suspension was initiated by only 2.0% sucrose supplementing the basal mineral salts of the medium. The list includes three types of saccharides (20 mono-, 10 di-, and 2 trisaccharides) in various forms: α and β, beta-D-(+), D-(−), and modifications such as methylated and non-hydrogenated.

*Amino acids*

The third group (Table S3) includes both groups of amino acids: the essential amino acids (leucine, phenylalanine, valine, and threonine), which cannot be synthesized by humans, and the non-essential amino acids (alanine, beta-alanine, asparagine, aspartic acid, proline, and serine), which are synthesized by both plants and humans.

*Amines*

The fourth group consisted primarily of amines, many of which are associated with sugar metabolism (Table S4).

*Polyhydric alcohols*

The fifth group comprises 14 polyhydric alcohols, including 4 linear-chain alcohols and 10 sugar alcohols (Table S5). The first subset of group 5 chemicals is characterized by a low retention time, i.e., propane-1,3-diol with a retention time of 6.162 min, while the second subset includes sugar alcohols with the highest retention time, up to 27.303 min. (e.g., galactinol). These data show the greatest difference in retention times among the described groups of organic compounds. The list includes sugar alcohols with a different number of hydroxyl groups: from two (propane-1,3-diol) to seven (galactinol).

*Nucleosides*

The fewest compounds were identified in the nucleoside group: inosine and uridine (Table S6).

*Sugar phosphates*

The seventh group includes 7 sugar phosphates, out of the 12 detected in the dataset between the various cycles of cell metabolism (Table S7).

*Others*

The eighth group comprises 13 organic compounds that may be involved in diverse metabolic pathways (Table S8).

Functional pathway enrichment analysis based on annotated metabolites revealed significant activation of both central and specialized metabolic routes. The most enriched pathways (FDR < 0.05) included glyoxylate and dicarboxylate metabolism, biosynthesis of unsaturated fatty acids, and biosynthesis of aromatic amino acids (phenylalanine, tyrosine, and tryptophan) (Table 1). These findings reflect a dynamic coordination among carbon assimilation, redox homeostasis, and precursor supply for the synthesis of secondary metabolites. Additional enrichment was detected for the pentose phosphate pathway, the TCA cycle, and carbon fixation in photosynthetic organisms, confirming high energy turnover and residual photosynthetic metabolic potential in *C. smithii* cells.

Furthermore, pathways related to the biosynthesis of flavonoids, stilbenoids, and alkaloids were statistically overrepresented (*p* < 0.05), supporting the metabolomic evidence for active secondary metabolism. Enrichment of galactose metabolism and amino/nucleotide sugar pathways indicates reinforcement of cell-wall biosynthesis and glycosylation processes. Together, these results suggest substantial metabolic versatility of *C. smithii* aggregates under in vitro conditions.

### 2.3. Analysis of Metabolites Measured at Five Time Points of the Cell Culture Duration

**Figure 2:**
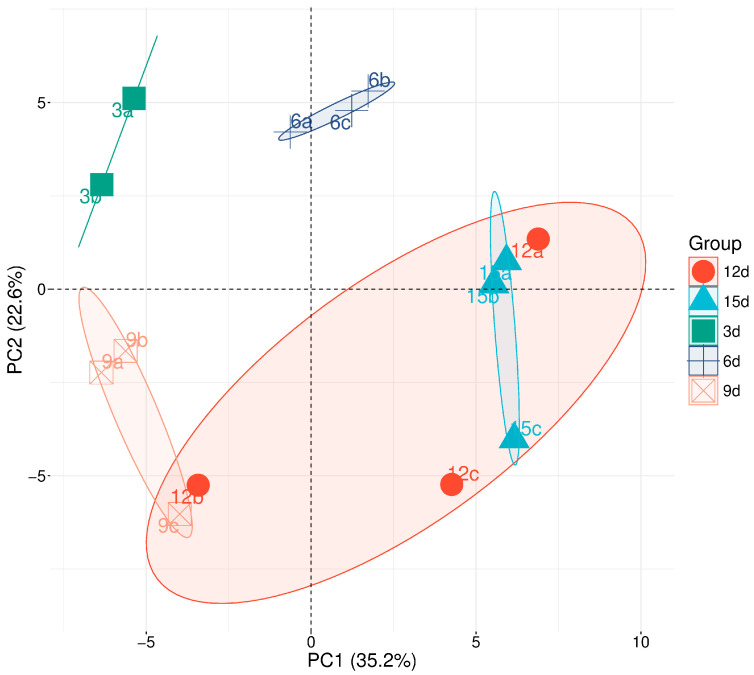
Principal component analysis (PCA) of the relative quantities of metabolites measured by GC/MS at different time points of the *Cyathea smithii* cell culture. The labels: 3d–15d indicate the sampling days: the 3rd, 6th, 9th, 12th, and 15th day after subculture; a–c indicate the technical repetitions.

A total of 138 major metabolites were identified in the *C. smithii* cell aggregates collected on days 3, 6, 9, 12, and 15 after subculture. The compounds were classified as primary or secondary metabolites. Principal Component Analysis (PCA) of their relative abundances revealed five distinct sample groups (Figure 2).

The first PCA group corresponds to samples collected during the early culture stage, immediately after subculture, representing the period of cellular adaptation to refreshed growth conditions. At this time point, the fresh weight was slightly lower than the initial mass of the inoculum (3 g per 80 mL of fresh medium).

The second PCA group includes samples harvested on days 6 and 9, representing two consecutive phases of active cell proliferation. Several newly synthesized metabolites were shared between these time points, reflecting similar metabolic activity during exponential growth. The third PCA group aligns with the transitional stage between rapid proliferation and the onset of metabolic slowdown. The fourth PCA group comprises the 12-day-old cultures, characterized by high metabolic activity accompanied by a gradual decline in proliferation. Microscopic observations revealed an accumulation of starch granules, which increased in size and number, as well as a general rise in cytoplasmic density (Figure 1). The fifth PCA group includes the samples collected on day 15, representing the late stationary phase of the culture, when metabolite profiles clearly diverged from earlier stages.

**Figure 3:**
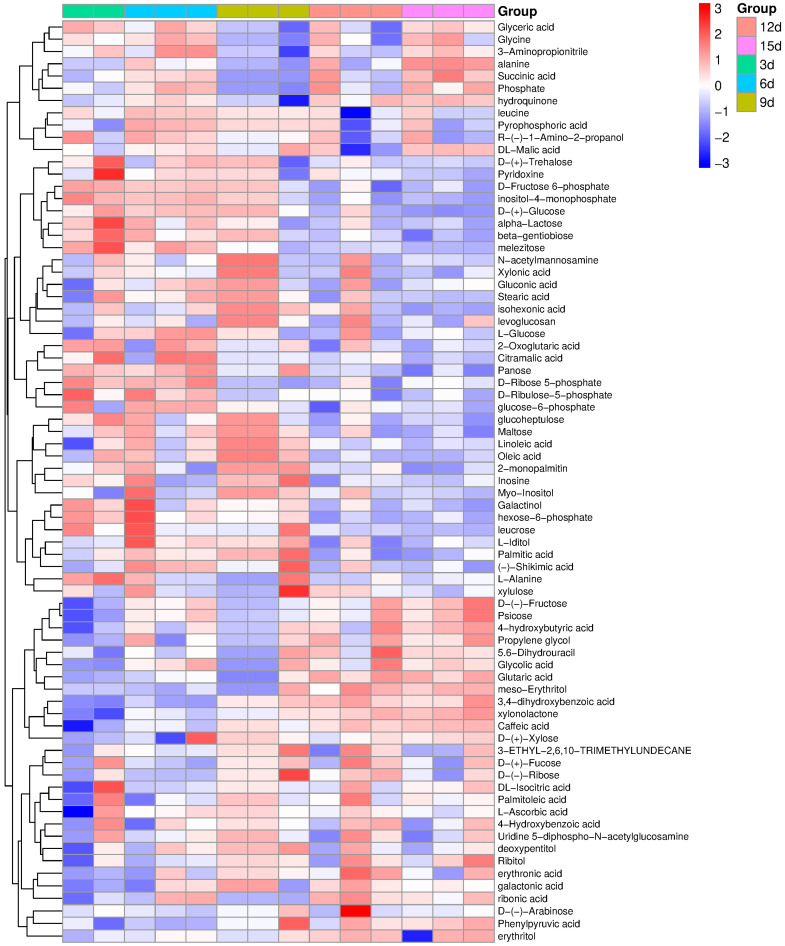
Heatmap illustrating the differentiation of metabolites across five sampling dates. Hierarchical clustering combined with pairwise *t*-tests (each vs. each) was applied. Red indicates a positive correlation, while blue indicates a negative correlation between metabolite abundance and sampling date. The labels 3d–15d indicate the sampling days: the 3rd, 6th, 9th, 12th, and 15th day after subculture.

Although PCA separated the samples into five well-defined groups, only 75 out of the 138 fully identified metabolites contributed significantly to this clustering (Figure 3). These metabolites are visualized in the heatmap, which separates into two major sections based on correlation patterns. The upper section shows low correlation among all sampling days, whereas the lower section reveals high correlation among metabolites from days 3, 6, and 9, with reduced correlation at later time points. These correlation trends are consistent with the typical growth curve of cell suspensions (Figure 1E), reflecting dynamic changes in the culture environment driven by gradual utilization of nutrients and metabolic reprogramming across development stages. The above description of the correlation between the metabolites is consistent with the growth curve, typical for the cell suspension, which was dynamically changing the culture environment through gradual utilization of the nutrient components of the medium (Figure 1E).

**Figure 4:**
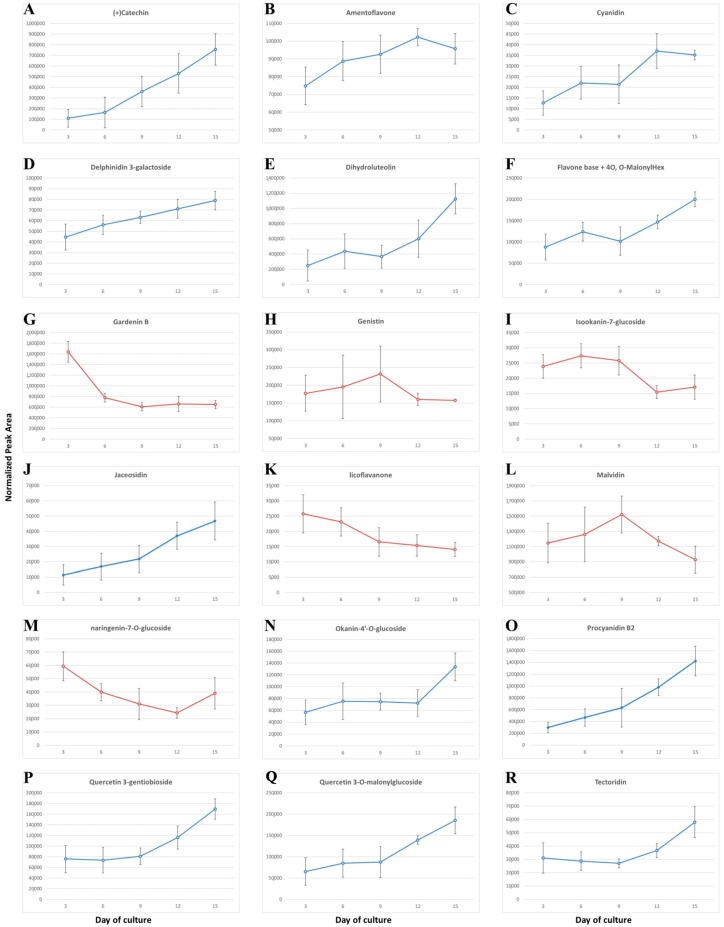
Changes in the content of selected flavonoids during the 15-day culture of *Cyathea smithii* in the presence of 2,4-D and BAP.

In the case of flavonoids, 12 compounds exhibited a consistent increase in their abundance over the 15-day culture period (Figure 4). Between the 3rd and 9th day, this increase was generally gradual, after which it became markedly more pronounced. Three flavonoids—gardenin B, licoflavanone, and naringenin-7-O-glucoside—displayed an opposite trend, with their concentrations gradually declining throughout the culture duration. Genistin, isocanin-7-glucoside, and malvidin showed biphasic accumulation patterns, characterized by an initial increase that reached a maximum around day 6 or 9 of culture, followed by a subsequent decrease.

## 3. Discussion

### 3.1. Primary Metabolites

Our metabolite identification system classified primary and secondary metabolites into eight chemically homogeneous groups, including flavonoids, and a ninth group (Table S8) comprising various unrelated compounds. Within this latter group, some compounds, such as arbutin or hydroquinone (restricted in the EU), are used in cosmetic production.

The 42 organic acids identified in the cell suspension (Table S1) highlight the central role of these compounds in the metabolism of both intact cells living in plant body and cells maintained in an unorganized structure under liquid culture conditions. The observed abundance of organic acids in our cell suspensions indicates that cells released from plant tissues maintain a broad spectrum of metabolic functions characteristic of intact plants. Organic acids are crucial in primary plant metabolism, participating in fundamental pathways not only related to carbon flux but also in plant adaptation to nutrient limitation, metal stress, and plant-microbe interactions at the root-soil interface [23].

As primary photosynthetic products, sugar alcohols (Table S5) contribute to stress responses, including low temperature, drought, and salinity, and may also enhance resistance to biotic stress. Suspension culture represents an artificial environment that can impose both biotic and abiotic stresses on the cells during subculture. Sugar alcohols are synthesized outside the chloroplast via reductases or a combination of reductases and phosphatases [24]. These alcohols serve diverse cellular functions in suspension cultures. For instance, galactinol and raffinose play protective roles against oxidative damage [25]. Galactinol synthase, which catalyzes the formation of galactinol from myo-inositol and UDP-galactose, is proposed to be the key enzyme controlling the biosynthesis of raffinose family oligosaccharides [25].

Amino acids are not only fundamental constituents of proteins but also serve as intermediates in numerous metabolic pathways. In cell culture, they are supplied individually in the growth medium to fulfill both structural and metabolic requirements. The concentration of amino acids in the medium changes dynamically due to their consumption by growing cells and the release of other amino acids during metabolism, making continuous monitoring an important part of culture optimization. Interestingly, biochemical analysis of the *C. smithii* cells did not reveal the complete set of both essential and non-essential amino acids. Out of eleven essential amino acids, only four were detected, in contrast to *Antenaria dioica*, in which the numbers of essential [9] and non-essential [8] amino acids were nearly equal in its aerial tissues [26]. This observation suggests either selective retention or metabolic transformation of certain amino acids in suspension-cultured cells, highlighting differences between in vitro cell aggregates and intact plant tissues.

The in vitro culture environment constantly presents unfavorable conditions that alter the physiology of plant tissues, and to survive in this harsh environment, the tissues must acclimate. Amino acids play an important role in maintaining the metabolism and growth of cultured cells under abiotic stress conditions. An example is proline, as studies indicate a positive relationship between proline accumulation and plant tolerance to various abiotic stresses [27] and in vitro organogenic and somatic embryo morphogenesis [28,29,30,31].

Under these conditions, amino acids play a pivotal role in maintaining cellular metabolism and supporting growth under abiotic stress. A prominent example is proline, whose accumulation has been positively correlated with plant tolerance to various abiotic stresses [27] and within in vitro organogenic and somatic embryogenesis processes [28,29,30,31]. Proline’s metal-chelating properties enable it to act as a reactive oxygen species scavenger, while its signaling functions activate specific genes essential for regeneration following stress [32]. Interestingly, proline was not detected among the amino acids accumulated in *C. smithii* cell suspensions. This suggests that, although proline accumulation is a highly conserved stress-response mechanism in plants, *C. smithii* cells may substitute proline with alternative compounds that fulfill comparable protective and regulatory roles in the in vitro environment.

The results of our study highlight the complexity of saccharide metabolism, particularly considering that only 2% sucrose is supplied during each culture passage. Sugars play a central role in plant growth and development, regulating protein synthesis and mRNA translation [33]. In the present analysis of *C. smithii* cell suspensions, 32 saccharides were identified, reflecting the intensive growth of cell aggregates, as evidenced by a three-and-a-half-fold increase in fresh biomass. Frequent cell divisions, cytoplasmic replication, continuous cell wall construction, and the high metabolic activity of the proliferating suspension are reflected in the accumulation of numerous mono-, di-, and trisaccharides (Table S2). The presence of phosphorylated forms of various sugars, predominantly 6C and 5C molecules, underscores metabolic changes associated with cellular energy demand (Table S7). The saccharide group, encompassing over 42 metabolites, includes 12 sugar phosphates, which serve as key intermediates in energy metabolism and provide carbon for most biosynthetic pathways in cells.

In plants, the hexose-phosphate pool, comprising interconvertible six-carbon sugars such as glucose-6-phosphate, glucose-1-phosphate, and fructose-6-phosphate, supplies carbon not only for glycolysis and the pentose phosphate pathway but also for cell wall and starch biosynthesis [34]. This was corroborated at the molecular level by the extensive formation of cell walls in actively dividing suspension cells. Additionally, inositol-4-monophosphate, like other inositols, contributes to cell growth and tissue differentiation, particularly through the formation of the middle lamella and the storage and transport of auxins [35].

The enrichment of several core carbon metabolic pathways, including the glyoxylate cycle, the TCA cycle, and the pentose phosphate pathway, highlights the metabolic flexibility of *C. smithii* cells maintained in suspension culture. The glyoxylate bypass likely serves a compensatory function during heterotrophic growth, providing a continuous supply of energy and carbon skeletons under conditions of reduced photosynthetic activity. Concurrently, the pentose phosphate pathway ensures the production of sufficient NADPH to support biosynthetic reactions and antioxidant defense, consistent with the observed accumulation of sugar phosphates and polyols in our metabolomic analysis.

The concurrent enrichment of unsaturated fatty acid biosynthesis and galactose metabolism suggests enhanced membrane lipid remodeling and active cell wall biosynthesis, processes characteristic of both dividing and differentiating plant cells in vitro. These pathways reflect a tightly regulated network that integrates primary metabolism, redox homeostasis, and cellular architecture maintenance during the growth of cell aggregates.

Table S6 lists only two nucleosides, inosine and uridine, detected in the cells of the studied suspension. Inosine, a nucleoside of hypoxanthine, serves as a precursor in the biosynthesis of AMP and GMP and as an intermediate in purine and purine nucleoside degradation. It also fulfills several physiological roles, including acting as a neuroprotective purine analog during purinergic signaling [36]. Uridine, a ribonucleoside containing uracil, contributes to carbohydrate metabolism through its phosphate derivatives and is a nucleotide base commonly found in foods such as beer, where it has been associated with enhanced cellular membrane synthesis and potentially cognitive benefits. It is noteworthy that only these two nucleosides were detected, despite the cells being actively dividing or later undergoing intensive secondary metabolite production. This observation may reflect limitations in detection sensitivity, the selective turnover of nucleosides in suspension culture, or rapid incorporation into nucleotides and other metabolites, highlighting the complexity of nucleotide metabolism under in vitro conditions.

### 3.2. Secondary Metabolites

Numerous studies have been focused on developing methods for the separation and identification of phenolic compounds in various matrices. Among these, liquid chromatography (LC) and gas chromatography (GC) coupled with mass spectrometry (MS) are the most widely used techniques [37]. While LC-MS does not require derivatization of compounds prior to analysis, in GC-MS phenolic compounds must be silylated. It has been shown that non-volatile and thermolabile phenolics are converted into volatile and thermotolerant trimethylsilyl derivatives through silylation [38]. Silyl derivatization has proven to be a reliable approach for the precise identification of phenolic compounds, allowing accurate quantification of a wide range of flavonoids. When combined with MS detection, this method provides a sensitive and accurate tool for both quantitative and qualitative analysis of flavonoids [39]. Both LC-MS and GC-MS are suitable for determining target phenolic metabolites; however, GC-MS is particularly advantageous for the quantitative analysis of compounds present at low concentrations [40]. Accordingly, we employed GC-MS to identify and screen phenolic metabolites in the cultured fern cells.

**Figure 5:**
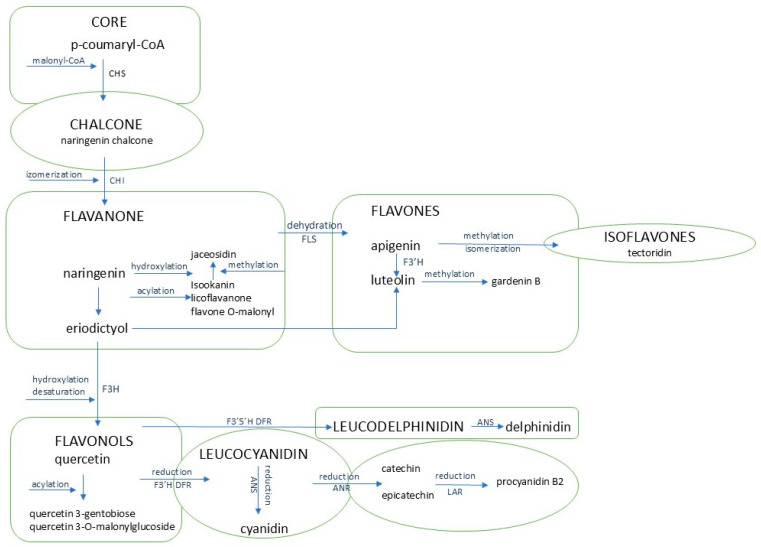
The diagram illustrates the correlation between specific groups of phenols, considering those found in the analyzed cell aggregates of the *Cyathea smithii* cell suspension.

Phenolic compounds [5], particularly flavonoids [41], which have also been reported in in vitro-derived *Cyathea delgadii* plants [42], possess a wide range of medicinal properties, including anticancer, anti-inflammatory, antiviral, neuroprotective, and cardioprotective activities, with demonstrated efficacy in multiple disease models. The biological effects of these compounds are influenced by the specific type of flavonoid and, notably, its bioavailability. This motivated our detailed investigation of these compounds as potential economical medicinal ingredients in fern cell cultures. Interest in these metabolites is further supported by the variable coloration of the culture medium observed in fern suspension cultures [12,13], which may reflect structural and functional diversification of phenolic compounds. Such diversity implies a broad spectrum of potential bioactivities, highlighting their relevance for disease prevention and therapeutic support. Figure 5 illustrates the interrelationships among the different groups of phenolic compounds identified in the culture.

The most representative group of flavonoids was identified as flavanones, particularly the flavone dihydroluteolin (eriodictyol) and gardenin B [43]. Flavan-3-ols, including catechin and its derivative procyanidin B2, were also prominently represented. Notably, the profile of dehydroleucodine, a substrate for multiple downstream derivatives, exhibited accumulation during cell suspension proliferation, with a kinetic pattern closely matching the cell growth curve. Similarly, the profiles of procyanidin B and its substrate, catechin [44], resembled those of dihydroluteolin and, importantly, reflected the dynamics of fresh and dry biomass during culture. In contrast, the profile of the methoxy derivative of dihydroluteolin, gardenin B, showed an opposite trend: its initially high concentration gradually decreased with culture age.

The most representative flavonoid in the suspension culture of *C. smithii* is the water-insoluble gardenin B. Future studies could focus on screening for other novel compounds arising from the conversion of naringenin or eriodictyol [45] under different environmental conditions, mediated by the enzymatic activities of F3′H and P450 reductase. Dihydroflavonones can be converted to flavonols through a reaction catalyzed by flavonol synthase (FLS) [46] or to leucocyanidin via dihydroflavonol reductase (DFR) [47]. Subsequently, leucocyanidin may be converted back to flavonols by anthocyanidin synthase (ANS) or transformed into catechin/epicatechin through the leucoanthocyanidin dioxygenase/leucoanthocyanidin reductase (LDOX/LAR) pathway, and ultimately, via anthocyanidin reductase (ANR), to produce procyanidin B2 [48].

Dimeric procyanidins (PAs) are formed when leucocyanidin, derived from catechin and/or epicatechin, attacks the nucleophilic C8 position of a catechin or epicatechin starter unit. The C8 position of the resulting dimer can then be attacked by a second leucocyanidin molecule, acting as an extender unit, producing oligomeric chains typically containing 4–8 linked units, which become increasingly insoluble as chain length increases. Interestingly, the assembly of PAs in plants may occur nonenzymatically, in contrast to the enzymatically regulated assembly of other major plant polymers such as cellulose, hemicellulose, and lignin. Within this class, quercetin derivatives, including quercetin-3-gentiobiose and quercetin-3-O-malonylglucoside, have also been identified [48,49].

Of all the flavonoids identified in the suspension culture of *C. smithii*, the most representative are the flavone gardenin B and the condensed tannin procyanidin B2. Gardenin B is practically insoluble in water, whereas procyanidin B2, in its dimeric form, is soluble up to 10 mg/mL. During cell culture growth, the accumulation of gardenin B decreases significantly, while the level of procyanidin B2 shows the opposite trend, increasing with culture duration. Almost all flavonoid subclasses were detected in the suspension culture. Among them, the most abundant flavanones were represented by eriodictyol [45], while gardenin B was the predominant flavone, and procyanidin B2 was the most abundant compound among condensed tannins. The initially high concentration of gardenin B, which declines as culture progresses, may indicate a role as a signal for the initiation of cell senescence. The concurrent increase in other flavonoid antioxidants may reflect an evolutionarily conserved cellular response, enhancing antioxidant capacity through the synthesis and accumulation of additional flavonoid antioxidants, potentially including condensed tannins. Gardenin B was first isolated from medicinal plants such as *Gardenia jasminoides, G. obtusifolia*, and *G. resinifera*, and has since been detected in several other species, including avocado [50]. In the present study, gardenin B was identified as an abundant flavone in *C. smithii* cell cultures. Most methoxyflavones have been isolated from citrus plants, though some are also found in other taxa, particularly Lamiaceae and Asteraceae [51]. Comprehensive profiling of polyphenols in avocado revealed numerous phenolic compounds, including gardenin B and the procyanidin B1 dimer in both ripe peel and flesh, with correlation analyses showing a significant association between these compounds and antioxidant activity [50]. Importantly, gardenin B isolated from *G. jasminoides* has been reported to act as a cell death inducer in human leukemia cells [43], and it has attracted considerable attention for its potential anticancer properties.

Procyanidin B2 is derived from epicatechin (Figure 5). Its polymerization is a non-enzymatic process, unlike the formation of other major plant polymers such as cellulose, hemicellulose, and lignin. Procyanidins accumulate in significant amounts during the early phase of seed coat formation, where they exhibit protective properties [52], acting against oxidative stress, herbivores (including insects and animals), and pathogen attack. They can also be incorporated into lignin structures; therefore, it can be hypothesized that procyanidins help protect the cell wall from damage under drought conditions, enabling plants to recover quickly when water becomes available again. Procyanidins are the most common type of proanthocyanidins, and their main constituent units are (+)-catechin and (−)-epicatechin, although (epi)-gallocatechin and (epi)-afzelchin units also occur. The profile of procyanidin B2 content during *C. smithii* cell growth showed two kinetic patterns: an initial slight increase followed by a significant accumulation during the period of intensive cell division. This period coincides with a marked decrease in the antioxidant gardenin B in the cell wall. We speculate that the increase in procyanidin B2 content may serve as a protective mechanism, helping cells counteract free radicals that accumulate during cell division and aging.

Secondary metabolites represented by epicatechin (Table S8), a flavan-3-ol, play an important antioxidant role in plants. However, other identified organic compounds also display noteworthy biological activities. For example, 3-aminopropionitrile exhibits anticancer properties by inhibiting the growth of breast adenocarcinoma cells [53]. Another compound, cynarin, is a natural product with a broad range of biological activities, including anti-HIV effects. It has recently been identified as a potent and highly selective HIV-1 integrase inhibitor [54]. The yellow color of the medium in the tested *C. smithii* cell suspension resembles that observed previously in the *C. delgadii* postculture medium. In the *C. delgadii* culture, among the identified flavonoids, particular attention was drawn to kaempferol-3-O-rutinoside, a bitter-tasting flavonol glycoside [13]. This compound was previously isolated from the rhizomes of the tropical herbaceous fern *Selliguea feei* [55].

The functional enrichment of pathways associated with phenylalanine, tyrosine, and tryptophan biosynthesis provides biochemical evidence for the activation of shikimate-derived secondary metabolism in *C. smithii*. These aromatic amino acids serve as direct precursors for flavonoid and alkaloid biosynthesis, both of which were strongly represented in our metabolomic dataset. In particular, the enrichment of the flavonoid biosynthesis pathway corroborates the chemical identification of gardenin B, procyanidin B, and other phenolic compounds. The presence of additional pathways, such as those involved in linoleic acid metabolism and stilbenoid biosynthesis, suggests a potential expansion of the fern’s chemical repertoire toward oxylipins and phenolic phytoalexins, which may contribute to its stress adaptation.

Collectively, these findings highlight a deep metabolic integration between energy-producing and specialized biosynthetic routes, demonstrating that *C. smithii* cell suspensions possess both the metabolic complexity and the flexibility characteristic of whole-plant systems.

## 4. Materials and Methods

### 4.1. Plant Material

The experiments were conducted on a cell suspension culture of *C. smithii*, established according to the procedure previously described by Rybczyński and co-workers [12]. The culture was maintained in half-strength MS medium (½ MS) [56] supplemented with 9.05 µM 2,4-D (dichlorophenoxyacetic acid) and 0.88 µM BAP (benzylaminopurine). The experiments were carried out in 250 mL conical flasks containing 80 mL of medium. Each subculture was initiated by transferring 3.0 g of tissue originating from the previous subculture. The flasks were plugged with cotton wool and covered with a protective paper cap. The cultures were maintained on a rotary horizontal shaker (Infors Rt 250, Bottmingen, Switzerland) at 120 rpm with a 3 cm amplitude. The cell suspensions were subcultured at two-week intervals using fresh medium. All cultures were grown under a 16/8 h day/night photoperiod of low-intensity diffuse light at 22 ± 1 °C in a phytotron. All in vitro manipulations were performed under sterile conditions in a laminar flow hood (Polon, Poznań, Poland).

### 4.2. Cell Suspension Sampling

Samples were collected at 3-day intervals (on the 3rd, 6th, 9th, 12th, and 15th day of culture). To maintain a constant culture volume, each sampling was followed by the replacement of an equal volume of fresh medium. The cultures were grown under a 16/8 h day/night, photoperiod at 22 ± 1 °C in a phytotron.

### 4.3. Preparation of Living and Fixed Specimens for Bright-Field Light Microscopy

Samples at various stages of culture development were isolated from the cell suspensions, and living specimens were examined using an Olympus AHBT-3 Vanox (Tokyo, Japan) bright-field light microscope equipped with Nomarsky differential interference contrast. For starch grain detection, the tissue samples were washed to remove the medium and subsequently fixed in FAA (formaldehyde:glacial acetic acid:ethanol; 5:5:90, *v*/*v*/*v*). After fixation, some of the cell aggregates were transferred to a microscope slide, stained with IKI (iodine in aqueous potassium iodide solution), gently warmed, and finally covered with a coverslip [12].

### 4.4. Metabolite Extraction from Cell Aggregates and Lc/Ms and Gc/Ms Metabolite Profiling

After completion of the growth period, the cell cultures were centrifuged, the culture medium was discarded, and the collected cells were frozen in liquid nitrogen. For each period of culture, cell samples were prepared in triplicate (from three independent conical flasks), with approximately 50 mg of material per sample, and stored at −80 °C for subsequent LC/MS and GC/MS analyses.

The frozen cell samples were ground in liquid nitrogen using precooled adapters for 45 s at 30 Hz frequency with a ball mill MM400 (Retsch, Haan, Germany). The resulting powder was extracted with 2 mL of 80% methanol. Ribitol (10 µL of a 1.0 mg/mL solution) was added as an internal standard. The suspensions were incubated in a microwave bath for 30 min. Samples were then centrifuged at 11,000× *g* at room temperature (RT) and evaporated at RT using a vacuum concentrator (Eppendorf, Hamburg, Germany).

Following drying over P_2_O_5_ under vacuum, each sample was resuspended in 100 µL of methoxyamine hydrochloride (20 mg/mL in dry pyridine) and vortex-mixed in a thermomixer for 1.5 h at 37 °C. The samples were briefly centrifuged (10 s) and supplemented with 160 µL of MSTFA (N-Methyl-N-(trimethylsilyl)trifluoroacetamide), followed by vortex-mixing in a thermomixer for 30 min at 37 °C. Samples were subsequently centrifuged at 11,000× *g* for 10 min. From each derivatized sample, 100 µL was transferred to a conical glass vial for GC analysis. Metabolites profiling was performed using LC/MS and GC/MS systems (TRACE 1310 GC oven with TSQ8000 triplequad MS from Thermo Fisher Scientific, Waltham, MA, USA) equipped with a DB-5MS column (30 m × 0.25 mm × 0.25 µm) (J &W Scientific, Agilent Technologies, Palo Alto, CA, USA). The GC temperature program was as follows: 70 °C for 2 min, ramped at 10 °C/min to 300 °C, and held at 300 °C for 10 min. Injector, interface, and source temperatures were set to 250 °C. The MS was operated in EI positive mode (electron energy 70 eV), scanning an *m*/*z* range of 50–850 [57]. Raw MS data were processed using the MSDial software package (v. 4.99). To correct retention time shifts (Rt) and to determine retention indices (RI), a standard alkane mixture (C-10–C-36) was injected into the GC/MS system. Identified artifacts (alkanes, column bleed, plasticizers, MSTFA derivatives, and reagents) were excluded from further analysis. Normalized data were exported to Excel for formatting and then used for statistical analyses.

Mass spectrometric measurements were performed using a timsTOF Pro instrument (Bruker Daltonics, Bremen, Germany) equipped with a conventional high-flow ESI source. Electrospray ionization was operated at ±4.5 kV, while nitrogen used both as the nebulizing gas (1.6 bar) and the drying gas, delivered at 8.0 L/min and 220 °C. External mass calibration was carried out using a sodium formate cluster solution, ensuring a mass accuracy better than 5 ppm. MS/MS data were acquired at a rate of 1 Hz, with precursor ions selected automatically from the MS spectra. Fragmentation energies, adjusted according to analyte molecular weights, ranged from 10 to 25 eV. Data acquisition was controlled using timsControl software (version 5.1.8), and subsequent data processing and evaluation were performed using DataAnalysis software (version 6.1, Bruker Daltonics).

## 5. Conclusions and Prospective Studies

In this study, a stable suspension of proliferating *C. smithii* cells was established. Chemical analyses revealed highly dynamic metabolic changes occurring in the cells during growth and senescence. A wide range of flavonoid-class compounds with potential biomedical relevance was detected. Notably, the ratio of gardenin B to procyanidin B may serve as an indicator of kinetic changes during cell proliferation in liquid culture.

The *C. smithii* suspension culture therefore offers both a model system for studying metabolism and stress responses in plant lineages and a promising platform for the biotechnological production of valuable secondary metabolites. Maintaining proliferating fern cells under controlled in vitro conditions provides a sustainable means of preserving genetic resources while enabling systematic exploration of their biochemical potential. The results presented here establish *C. smithii* as a promising source of novel natural products with antioxidant, anti-inflammatory, and anticancer potential while also contributing to the conservation and functional understanding of an evolutionarily ancient plant lineage.

Given the increasing demand for novel bioactive compounds, future studies should aim to correlate metabolite profiles with specific biological activities and investigate how external factors (e.g., light, electromagnetic field, or epigenetic modifiers) influence metabolism pathways. They should integrate metabolite data with transcriptomic and proteomic analyses to uncover the enzymatic pathways responsible for flavonoid biosynthesis and to understand their regulation under changing environmental conditions.

## Figures and Tables

**Table 1:** Functional pathway enrichment of the identified metabolites based on GC-MS and LC-MS data.

	Total	Expected	Hits	Raw *p*	-LOG10(p)	Holm Adjust	FDR	Impact
Glyoxylate and dicarboxylate metabolism	29	1.1342	6	0.00065019	3.187	0.062418	0.038884	0.30155
Biosynthesis of unsaturated fatty acids	22	0.86042	5	0.0012151	2.9154	0.11544	0.038884	0
Phenylalanine, tyrosine and tryptophan biosynthesis	22	0.86042	5	0.0012151	2.9154	0.11544	0.038884	0.18018
Galactose metabolism	27	1.056	5	0.0032043	2.4943	0.298	0.076904	0.2175
Pentose phosphate pathway	19	0.74309	4	0.0052643	2.2787	0.48431	0.091485	0.37439
Citrate cycle (TCA cycle)	20	0.7822	4	0.0063903	2.1945	0.58152	0.091485	0.26155
Carbon fixation in photosynthetic organisms	21	0.82131	4	0.0076663	2.1154	0.68996	0.091485	0.07173
Flavonoid biosynthesis	47	1.8382	6	0.0084883	2.0712	0.75546	0.091485	0.19444
Linoleic acid metabolism	4	0.15644	2	0.0085767	2.0667	0.75546	0.091485	1
Amino sugar and nucleotide sugar metabolism	50	1.9555	6	0.011467	1.9406	0.9976	0.11008	0
Pentose and glucuronate interconversions	16	0.62576	3	0.022128	1.6551	1	0.19311	0.34375
Aminoacyl-tRNA biosynthesis	46	1.7991	5	0.030811	1.5113	1	0.24648	0
Tropane, piperidine and pyridine alkaloid biosynthesis	8	0.31288	2	0.036175	1.4416	1	0.24806	0
Stilbenoid, diarylheptanoid and gingerol biosynthesis	8	0.31288	2	0.036175	1.4416	1	0.24806	0.2647

## Data Availability

The original contributions presented in this study are included in this article/Supplementary Materials. Further inquiries can be directed to the corresponding authors.

## Supplementary Materials

The following supporting information can be downloaded at: https://www.mdpi.com/article/10.3390/ijms262311683/s1.

## Abbreviations

The following abbreviations are used in this manuscript:

BAP	6-Benzylaminopurine
2,4-D	Dichlorophenoxyacetic acid
GC/MS	Gas Chromatography/Mass Spectroscopy
LC/MS	Liquid Chromatography/Mass Spectroscopy
MS	Murashige and Skoog medium
NAA	Naphthalene Acetic Acid
PCA	Principal Component Analysis
TDZ	Thidiazuron (1-Phenyl-3-(1,2,3-thiadiazol-5-yl)urea
