# Supplementary material for: Cell Suspension of the Tree Fern Cyathea smithii (J.D. Hooker) and Its Metabolic Potential During Cell Growth: Preliminary Studies
Source: Int J Mol Sci. 2025 Dec 2;26(23):11683. doi: 10.3390/ijms262311683 (PMC12692112; doi:10.3390/ijms262311683)
Supplement: Supplementary file 1 [file ijms-26-11683-s001.zip › ijms-3899374-supplementary.pdf]

Supplementary Materials

Cell Suspension of the Tree Fern *Cyathea smithii* (J.D. Hooker) and Its Metabolic Potential During Cell Growth: Preliminary Studies

Jan J. Rybczyński [1,*], Łukasz Marczak [2], Katarzyna Skórkowska-Telichowska [3,4], Maciej Stobiecki [2], Jan Szopa [5] and Anna Mikuła [1,*]

Table S1. The list of acids.

No.	Name	Average Rt(min)	Average RI	Quant mass
1.	Threonic acid	13.127	1548.96	73.0142
2.	Tartaric acid	13.608	1587.13	73.00925
3.	Succinic acid	9.955	1308.45	147.0692
4.	Stearic acid	20.25	2235.89	117.0039
5.	(-)-Shikimic acid	15.993	1796.74	204.0738
6.	Quinic acid	16.564	1852.53	83.06134
7.	Pyrophosphoric acid	9.255	1258.2	73.02838
8.	Phenylpyruvic acid	13.497	1578.57	73.0267
9.	Palmitoleic acid	18.337	2029.52	74.99534
10.	Palmitic acid	18.437	2040.06	117.0086
11.	2-Oxoglutaric acid	13.334	1565.31	73.01907
12.	Oleic acid	20.017	2208.93	75.00332
13.	Nicotinic acid	9.755	1294.56	110.0167
14.	Malonic acid	8.299	1187.38	147.0973
15.	Malic acid	12.255	1479.17	424.1978
16.	Linoleic acid	19.963	2202.52	75.00545
17	Xylonic acid	15.472	1750.9	73.0118
18.	L-Ascorbic acid	16.47	1842.88	157.0625
19.	Lactobionic acid	24.317	2740.52	204.0676
20.	L-(+)-Tartaric acid	14.046	1624.4	147.0564
21.	L-(-)-Malic acid	12.258	1479.98	73.005
22.	Isothreonic acid	12.921	1532.6	73.01669
23.	Isohexonic acid	18.077	2001.78	73.01226
24.	Glycolic acid	6.863	1043.55	147.0941
25.	Glyceric acid	10.153	1322.48	73.02389
26.	Glutaric acid	11.509	1420.31	201.1588
27.	Gluconic acid	17.939	1988.08	72.99781
28.	Galactonic acid	17.867	1980.9	73.01527
29.	Erythronic acid	13.65	1590.32	73.00768
30.	DL-Malic acid	12.336	1486.1	305.1132

31.	DL-Isocitric acid	16.101	1807.26	103.0383
32.	Citric acid	16.077	1805.49	147.051
33.	Citramalic acid	12.039	1462.63	73.02386
34.	cis-Aconitic acid	15.336	1738.78	147.0643
35.	Caffeic acid	19.273	2129.58	219.0219
36.	5-Keto-D-Gluconic acid	16.673	1861.52	103.0079
37.	4-hydroxybutyric acid	8.85	1230.42	147.0868
38.	GABA (gamma amino- butric acid)	12.806	1523.94	117.0472
39.	3-Hydroxypropionic acid	7.767	1134.26	147.0937
40.	3.4-Dihydroxybenzoic acid	16.12	1808.77	193.0174
41.	2-Hydroxyisobutyric acid	7.21	1078.35	131.0897
42.	Xylonic acid	15.472	1750.9	73.0118

Table S2. The list of saccharides.

No.	Name	Average Rt(min)	Average RI	Quant mass
1.	Psicose (mono)*	16.723	1869.02	72.96339
2.	Methylhexose (mono)	16.062	1803.44	204.0605
3.	1-methylgalactose (mono)	17.341	1927.02	204.025
4.	L-Glucose (mono)	17.158	1909.66	162.0862
5.	Hexose (mono)	17.929	1986.56	72.06839
6.	Erythrose (mono)	11.791	1443.18	205.109
7.	2-Deoxy-D-glucose (mono)	15.81	1780.58	147.0617
8.	D-Xylulose (mono)	14.233	1641.59	244.0563
9.	D-(+)-Xylose (mono)	14.163	1634.57	191.0629
10.	D-(+)-Mannose (mono)	16.397	1836.2	73.01416
11.	D-(+)-Glucose (mono)	17.685	1962.73	281.0579
11.	D-(+)-Fucose (mono)	14.965	1705.69	117.0415
13.	D-(-)-Ribose (mono)	14.514	1665.83	73.008
14.	D-(-)-Fructose (mono)	16.647	1860.19	216.0373
15.	D-(-)-Arabinose (mono)	14.339	1650.41	103.0509
16.	beta-D-(+)-Glucose (mono)	17.16	1911.36	72.98964
17.	Allose (mono)	17.014	1897.15	116.0377
18.	3.6-anhydro-D-glucose (mono)	15.366	1741.23	73.01228
19.	1.6-anhydro-beta-D-glucose (mono)	14.588	1672.36	204.0825
20.	Xylulose (mono)	14.504	1664.43	173.0719
21.	D-(+)-Trehalose (di)	24.213	2727.26	361.155
22.	Sucrose (di)	23.023	2568.11	217.0485
23.	Sophorose (di)	24.444	2757.63	204.0868
24.	Leucrose (di)	22.922	2555.79	204.0649
25.	Glucoheptulose (di)	21.216	2347.54	73.01044
26.	alpha-Lactose (di)	22.389	2489.09	204.0665

27.	Melibiose (di)	25.138	2849.16	204.0494
28.	Maltose (di)	24.374	2747.75	204.063
29.	D-(+)-Maltose (di)	24.188	2722.39	187.8245
30.	beta-Gentiobiose (di)	24.382	2749.49	160.0721
31.	Panose (tri)	33.8	3749.44	361.0992
32.	Melezitose (tri)	30.933	3461.61	217.0593

* Mono-, di-, tri- saccharides

Table S3. The list of amino acids.

No.	Name	Essentiality	Average Rt (min)	Average RI	Quant mass
1.	Alanine	NEAA	10.568	1351.77	188.1215
2.	L-Alanine	NEAA	6.857	1042.99	116.1028
3.	L-Aspartic acid	NEAA	12.643	1510.66	52.94909
4.	L-Asparagine	NEAA	14.439	1659.61	260.984
5.	Leucine	EAA	9.27	1261.12	73.01326
6.	L-Serine	NEAA	11.129	1391.35	147.0637
7.	N-Acetyl-DL-serine	NEAA	12.504	1499.13	186.0499
8.	L-5-Oxoproline	NEAA	12.678	1513.3	156.0677
9.	Phenylalanine	EAA	13.097	1545.86	120.0541
10.	Threonine	EAA	9.682	1289.01	117.086
11	L-Valine	EAA	8.534	1208.11	144.1476

EAA – essential amino acids: NEAA – non-essential amino acids

Table S4. The list of amines.

No.	Name	Average Rt (min)	Average RI	Quant mass
1.	Uridine 5'-diphospho-N-acetylglucosamine	16.19	1816.17	103.0291
2.	N-acetylmannosamine	18.977	2097.57	73.01453
3.	N-acetyl-d-hexosamine	19.248	2126.31	73.0079
4.	N-Acetyl-D-glucosamine	18.784	2077.56	204.0581
5.	N-Acetyl-D-galactosamine	19.203	2121.35	210.8948
6.	Hydroxylamine	7.035	1060.71	73.06095
7.	Ethanolamine	9.197	1254.57	299.2108
8.	Diethanolamine	11.384	1410.93	218.1656
9.	D-(+)-Glucosamine	17.283	1923.75	319.1412
10.	D-(+)-Galactosamine	17.496	1944.35	299.2158
11.	Uridine 5'-diphospho-N-acetylglucosamine	16.19	1816.17	103.0291

Table S5. The list of polyhydric alcohols (sugar alcohols).

No.	Name	Average Rt (min)	Average RI	Quant mass
1.	R-(-)-1-Amino-2-propanol	9.91	1305.23	174.1075
2.	Propylene glycol	6.225	979.51	73.05737
3.	Propane-1,3-diol	6.162	973.09	147.0862
4.	Myo-Inositol	18.829	2081.72	72.9914
5.	meso-Erythritol	12.491	1498.41	73.02053
6.	Maltitol	24.945	2824.41	217.1206
7.	L-Iditol	17.599	1954.52	361.1368
8.	Lactitol	24.447	2758.39	319.1198
9.	Inositol	18.846	2083.53	337.9876
10.	Glycerol	9.381	1267.85	147.0672
11.	Galactitol	17.975	1991.79	175.0468
12.	Galactinol	27.303	3098.85	204.0617
13.	Erythritol	12.496	1499.08	147.0768
14.	Peroxypentitol	13.814	1603.89	73.00991

Table S6. The list of nucleosides.

No.	Name	Average Rt(min)	Average RI	Quant mass
1.	Inosine	23.077	2574.93	73.01486
2.	Uridine	21.952	2435.1	217.0732

Table S7. The list of phosphates.

No.	Name	Average Rt(min)	Average RI	Quant mass
1.	Phosphate	9.361	1265.54	147.0379
2.	Methanol phosphate	7.959	1153.64	241.084
3.	Inositol-4-monophosphate	21.653	2397.95	147.0449
4.	Hexose-6-phosphate	21.422	2371.35	204.0585
5.	Glucose-6-phosphate	20.852	2305.95	273.9748
6.	Glucose-1-phosphate	15.922	1790.46	73.0157
7.	Ethanol phosphate	8.649	1216.18	210.9955
8.	D-Ribulose-5-phosphate	19.048	2105.04	357.089
9.	D-Ribose 5-phosphate	18.947	2094.23	299.0523
10.	D-Glucose 6-phosphate	21.003	2322.96	387.0683
11.	D-Fructose 6-phosphate	20.734	2291.31	73.0183
12.	rac-Glycerol 3-phosphate	15.457	1749.29	299.0519

Table S8. The list of other compounds.

No.	Name	Average Rt (min)	Average RI	Quant mass	
1.	beta-Mannosylglycerate	20.142	2223.28	204.0691	glicerate
2.	Arbutin	22.981	2562.96	275.1003	glicoside
3.	3-ethyl-2.6.10-Trimethylundecane	13.722	1596.22	57.05369	alkane
4.	3-Aminopropionitrile	10.459	1344.18	73.02103	nitrile
5.	2-Monoolein	24.05	2704.08	103.0136	oleic acid
6.	2-Monopalmitin	22.805	2541.07	129.0166	It derives from an oleic acid
7.	1-Monopalmitin	23.066	2573.27	371.2685	It derives from an oleic acid
8.	1,3-Dihydroxyacetone dimer	9.061	1245.29	73.04227	aceton
9.	glycerol-3-galactoside	20.796	2298.92	204.0582	galactoside
10.	Hydroquinone	11.176	1394.97	239.1012	phenolic
11.	methyl O-D-Galactopyranoside	16.058	1801.59	73.02015	galactopyranan
12.	Levoglucosan	14.821	1692.98	73.01627	carbohydrate
13.	Xylonolactone	13.754	1598.82	217.0933	lactone
